# *catena*-Poly[[diaquacobalt(II)]-bis(*μ*-3-carboxy­adamantane-1-carboxyl­ato-κ^2^ *O* ^1^:*O* ^3^)]

**DOI:** 10.1107/S1600536809007387

**Published:** 2009-03-06

**Authors:** Li-Ming Tang, Jia-Hui Xu, Xiao-Yan Han, Wei Xu

**Affiliations:** a State Key Laboratory Base of Novel Functional Materials and Preparation Science, Faculty of Materials Science and Chemical Engineering, Institute of Solid Materials Chemistry, Ningbo University, Ningbo, Zhejiang, 315211, People’s Republic of China

## Abstract

In the title compound, [Co(C_12_H_15_O_4_)_2_(H_2_O)_2_]_*n*_, adjacent Co^II^ atoms (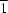 symmetry) are bridged by 3-carboxy­adamantane-1-carboxyl­ate anions, forming a chain running along [001]. Inter­chain O—H⋯O hydrogen bonds link the chains into layers parallel to (100); the layers are further connected *via* inter­layer hydrogen bonds inter­actions, forming a three-dimensional framework.

## Related literature

For related compounds, see: Nielsen *et al.* (2008 ▶); Zhao *et al.* (2007 ▶); Zheng *et al.* (2008 ▶).
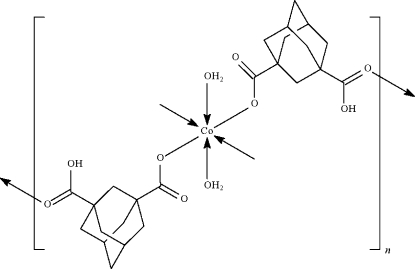


## Experimental

### 

#### Crystal data

[Co(C_12_H_15_O_4_)_2_(H_2_O)_2_]
*M* *_r_* = 541.44
Orthorhombic, 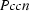

*a* = 10.718 (2) Å
*b* = 23.638 (5) Å
*c* = 9.0726 (18) Å
*V* = 2298.6 (8) Å^3^
*Z* = 4
Mo *K*α radiation
μ = 0.81 mm^−1^
*T* = 293 K
0.10 × 0.10 × 0.10 mm

#### Data collection

Rigaku R-AXIS RAPID diffractometer
Absorption correction: multi-scan (*ABSCOR*; Higashi, 1995 ▶) *T*_min_ = 0.921, *T*_max_ = 0.925
20865 measured reflections
2622 independent reflections
2145 reflections with *I* > 2σ(*I*)
*R*_int_ = 0.033

#### Refinement

*R*[*F* ^2^ > 2σ(*F* ^2^)] = 0.031
*wR*(*F* ^2^) = 0.086
*S* = 1.06
2622 reflections
161 parameters
H-atom parameters constrained
Δρ_max_ = 0.35 e Å^−3^
Δρ_min_ = −0.30 e Å^−3^

### 

Data collection: *RAPID-AUTO* (Rigaku, 1998 ▶); cell refinement: *RAPID-AUTO*; data reduction: *CrystalStructure* (Rigaku/MSC, 2004 ▶); program(s) used to solve structure: *SHELXS97* (Sheldrick, 2008 ▶); program(s) used to refine structure: *SHELXL97* (Sheldrick, 2008 ▶); molecular graphics: *SHELXTL* (Sheldrick, 2008 ▶); software used to prepare material for publication: *SHELXL97*.

## Supplementary Material

Crystal structure: contains datablocks ptcLa, I. DOI: 10.1107/S1600536809007387/ng2552sup1.cif

Structure factors: contains datablocks I. DOI: 10.1107/S1600536809007387/ng2552Isup2.hkl

Additional supplementary materials:  crystallographic information; 3D view; checkCIF report

## Figures and Tables

**Table 1:** Selected bond lengths (Å)

Co—O1	2.0574 (12)
Co—O5	2.0956 (14)
Co—O4^i^	2.1061 (12)

Symmetry code: (i) 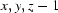.

**Table 2:** Hydrogen-bond geometry (Å, °)

*D*—H⋯*A*	*D*—H	H⋯*A*	*D*⋯*A*	*D*—H⋯*A*
O3—H1⋯O1^ii^	0.81	1.82	2.6058 (19)	166
O5—H2⋯O2	0.80	2.07	2.7762 (18)	147
O5—H3⋯O2^iii^	0.81	2.02	2.8334 (18)	175

Symmetry codes: (ii) 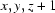; (iii) 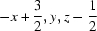.

**Fig. 1.**
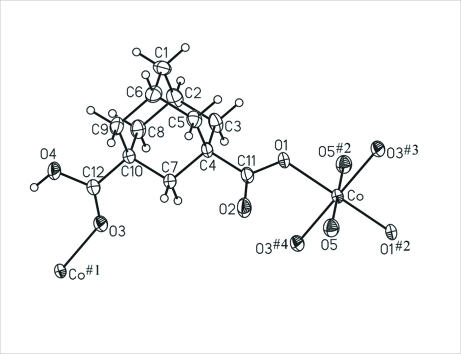
View of the molecular structure of the title compound, Displacement ellipsoids are drawn at the 45% probability level.[Symmetry codes: (i) x, y, z + 1; (ii) -x + 1, -y + 1, -z; (iii) x, y, z - 1; (iv) -x + 1, -y + 1, -z + 1.]

**Fig. 2.**
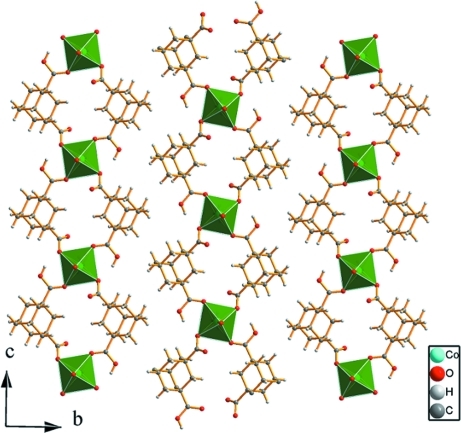
The two-dimensional layer structure constructed by one-dimensional chains through hydrogen bonds interaction (the hydrogen bonds are neglected)

## supplementary crystallographic information

### Comment

The cambridge Structural Database (Version 5.30, February 2009) lists few examples of metal (II) adamantane-1,3-dicarboxylates (Nielsen *et al.*, 2008; Zhao *et al.*, 2007). The dicarboxylate group is rigid, much more different from the aliphatic dicarboxylic acids (Zheng *et al.*, 2008), effected severely by spacial steric hindrance. The asymmetric unit of the title compoud consists of one Co^2+^ cation, one aqua ligand and one Hadc^-^ anion (H_2_adc = adamantane-1,3-dicarboxylic acid) (Fig.1). The Co atoms at the Wckoff 4a sites are crystallographically imposed by iversion center and are each located in an elongated octahedral coordination sphere defined by two aqua ligands and four carboxylate oxygen atoms from four 3-carboxyadamantane-1-carboxylate anions. The axial Co—O bond distances averaged at 2.106 (1) Å are slightly longer than the equatorial ones of 2.078 (1) Å. The *trans*- and *cisoid* O—Co—O angles fall in the regions 88.49 (5)–91.51 (5)° and 180°, respectively, exhibiting slight diviation from the corresponding values for a regular geometry (Table 1). Each carboxylate anion monodentately coordinates one Co^2+^ ion in *syn* fashion. Interestingly, one of the two carboxylate anions from each ligand is protonated and coordinates one Co^2+^ ion by carbonyl oxygen atom, which is rare in former reports. The Co^2+^ ions are bridged by 3-carboxyadamantane-1-carboxylate anions to form one-dimensional chains running along the [001] direction. On the basis of the interchain O—H···O hydrogen bonds (Table 2),these chains are assembled into layers parallel to (100) (Fig.2). The layers are further connected to form a three-dimensional framework *via* interlayer hydrogen bonds interaction.

### Experimental

Adamantane-1,3-dicarboxylic acid (H_2_adc) (0.0666 g, 0.3 mmol), 1 *M* NaOH (0.5 ml, 0.5 mmol) was consequently added to 15 mol aqueous solution, then the mixture was heated constantly at 90 °C and stirred for 30 min, yielding colorless solution, to which was added CoCl_2_.6H_2_O (0.2485 g, 1.0 mmol) and continually stirred for 30 min, then the purple solution (pH = 5.12) was cooled to room temperature and laid undisturbed, purple block-like crystals was afforded after two weeks.

### Refinement

H atoms bonded to C atoms were palced in geometrically calculated position and were refined using a riding model, with *U*_iso_(H) = 1.2 *U*_eq_(C). H atoms attached to O atoms were found in a difference Fourier synthesis and were refined using a riding model, with the O—H distances fixed as initially found and with *U*_iso_(H) values set at 1.2 *U*eq(O).

### Crystal data

**Table:**

[Co(C_12_H_15_O_4_)_2_(H_2_O)_2_]	*F*(000) = 1140
*M**_r_* = 541.44	*D*_x_ = 1.565 Mg m^−^^3^
Orthorhombic, *P**c**c**n*	Mo *K*α radiation, λ = 0.71073 Å
Hall symbol: -P 2ab 2ac	Cell parameters from 20865 reflections
*a* = 10.718 (2) Å	θ = 3.1–27.5°
*b* = 23.638 (5) Å	µ = 0.81 mm^−^^1^
*c* = 9.0726 (18) Å	*T* = 293 K
*V* = 2298.6 (8) Å^3^	Block, purple
*Z* = 4	0.10 × 0.10 × 0.10 mm

### Data collection

**Table:**

Rigaku R-AXIS RAPID diffractometer	2622 independent reflections
Radiation source: fine-focus sealed tube	2145 reflections with *I* > 2σ(*I*)
graphite	*R*_int_ = 0.033
Detector resolution: 0 pixels mm^-1^	θ_max_ = 27.5°, θ_min_ = 3.1°
ω scans	*h* = −13→13
Absorption correction: multi-scan (*ABSCOR*; Higashi, 1995)	*k* = −30→30
*T*_min_ = 0.921, *T*_max_ = 0.925	*l* = −11→11
20865 measured reflections	

### Refinement

**Table:**

Refinement on *F*^2^	Primary atom site location: structure-invariant direct methods
Least-squares matrix: full	Secondary atom site location: difference Fourier map
*R*[*F*^2^ > 2σ(*F*^2^)] = 0.031	Hydrogen site location: inferred from neighbouring sites
*wR*(*F*^2^) = 0.086	H-atom parameters constrained
*S* = 1.06	*w* = 1/[σ^2^(*F*_o_^2^) + (0.0444*P*)^2^ + 0.9177*P*] where *P* = (*F*_o_^2^ + 2*F*_c_^2^)/3
2622 reflections	(Δ/σ)_max_ = 0.001
161 parameters	Δρ_max_ = 0.35 e Å^−^^3^
0 restraints	Δρ_min_ = −0.30 e Å^−^^3^

### Special details

**Table:**

Geometry. All e.s.d.'s (except the e.s.d. in the dihedral angle between two l.s. planes) are estimated using the full covariance matrix. The cell e.s.d.'s are taken into account individually in the estimation of e.s.d.'s in distances, angles and torsion angles; correlations between e.s.d.'s in cell parameters are only used when they are defined by crystal symmetry. An approximate (isotropic) treatment of cell e.s.d.'s is used for estimating e.s.d.'s involving l.s. planes.
Refinement. Refinement of *F*^2^ against ALL reflections. The weighted *R*-factor *wR* and goodness of fit *S* are based on *F*^2^, conventional *R*-factors *R* are based on *F*, with *F* set to zero for negative *F*^2^. The threshold expression of *F*^2^ > σ(*F*^2^) is used only for calculating *R*-factors(gt) *etc*. and is not relevant to the choice of reflections for refinement. *R*-factors based on *F*^2^ are statistically about twice as large as those based on *F*, and *R*- factors based on ALL data will be even larger.

### Fractional atomic coordinates and isotropic or equivalent isotropic displacement parameters (Å^2^)

**Table:**

	*x*	*y*	*z*	*U*_iso_*/*U*_eq_	
Co	0.5000	0.5000	0.0000	0.02148 (11)	
C1	0.50924 (15)	0.62852 (7)	0.64285 (18)	0.0239 (3)	
C2	0.37038 (16)	0.64210 (7)	0.61757 (18)	0.0300 (4)	
H2A	0.3213	0.6077	0.6246	0.036*	
H2B	0.3413	0.6683	0.6924	0.036*	
C3	0.35442 (17)	0.66835 (9)	0.46523 (19)	0.0343 (4)	
H3A	0.2660	0.6770	0.4490	0.041*	
C4	0.39899 (16)	0.62682 (8)	0.34654 (18)	0.0310 (4)	
H4A	0.3497	0.5924	0.3511	0.037*	
H4B	0.3878	0.6435	0.2498	0.037*	
C5	0.53696 (15)	0.61264 (7)	0.37055 (16)	0.0212 (3)	
C6	0.55413 (17)	0.58683 (7)	0.52439 (16)	0.0256 (3)	
H6A	0.6415	0.5780	0.5403	0.031*	
H6B	0.5069	0.5519	0.5315	0.031*	
C7	0.4296 (2)	0.72253 (8)	0.4553 (2)	0.0394 (5)	
H7A	0.4012	0.7490	0.5297	0.047*	
H7B	0.4176	0.7398	0.3593	0.047*	
C8	0.56719 (19)	0.70952 (7)	0.47855 (19)	0.0327 (4)	
H8A	0.6155	0.7446	0.4718	0.039*	
C9	0.58580 (17)	0.68311 (7)	0.63123 (18)	0.0295 (4)	
H9A	0.5594	0.7096	0.7068	0.035*	
H9B	0.6735	0.6748	0.6465	0.035*	
C10	0.61268 (16)	0.66778 (7)	0.36080 (18)	0.0278 (4)	
H10A	0.7005	0.6597	0.3758	0.033*	
H10B	0.6028	0.6844	0.2637	0.033*	
C11	0.58484 (15)	0.57136 (7)	0.25371 (16)	0.0247 (3)	
O1	0.52461 (12)	0.56998 (5)	0.13165 (12)	0.0296 (3)	
O2	0.67854 (12)	0.54217 (6)	0.27703 (13)	0.0378 (3)	
C12	0.51789 (16)	0.60130 (7)	0.79318 (18)	0.0280 (4)	
O3	0.51926 (19)	0.63717 (6)	0.90404 (15)	0.0615 (5)	
H1	0.5154	0.6209	0.9823	0.074*	
O4	0.51779 (12)	0.55044 (5)	0.80980 (13)	0.0312 (3)	
O5	0.69310 (12)	0.48644 (6)	0.00823 (12)	0.0314 (3)	
H2	0.7197	0.5019	0.0807	0.038*	
H3	0.7333	0.5010	−0.0575	0.038*	

### Atomic displacement parameters (Å^2^)

**Table:**

	*U*^11^	*U*^22^	*U*^33^	*U*^12^	*U*^13^	*U*^23^
Co	0.02775 (18)	0.02296 (17)	0.01371 (17)	0.00182 (11)	0.00009 (11)	0.00032 (11)
C1	0.0317 (9)	0.0242 (7)	0.0158 (7)	0.0027 (6)	0.0009 (6)	0.0004 (6)
C2	0.0277 (9)	0.0389 (9)	0.0232 (8)	−0.0011 (7)	0.0058 (7)	−0.0017 (7)
C3	0.0239 (9)	0.0516 (11)	0.0274 (8)	0.0131 (8)	−0.0004 (7)	0.0010 (8)
C4	0.0250 (9)	0.0467 (10)	0.0213 (8)	0.0020 (7)	−0.0039 (6)	−0.0009 (7)
C5	0.0233 (7)	0.0267 (7)	0.0137 (6)	0.0007 (6)	−0.0009 (6)	−0.0011 (6)
C6	0.0361 (9)	0.0243 (7)	0.0164 (7)	0.0041 (7)	0.0003 (6)	−0.0009 (6)
C7	0.0554 (13)	0.0336 (9)	0.0292 (9)	0.0186 (9)	0.0036 (9)	0.0067 (8)
C8	0.0438 (11)	0.0246 (8)	0.0296 (9)	−0.0059 (7)	0.0065 (8)	0.0010 (7)
C9	0.0349 (9)	0.0297 (8)	0.0239 (8)	−0.0043 (7)	−0.0014 (7)	−0.0066 (7)
C10	0.0303 (9)	0.0308 (8)	0.0222 (8)	−0.0032 (7)	0.0047 (6)	0.0028 (7)
C11	0.0298 (8)	0.0283 (7)	0.0160 (7)	−0.0005 (6)	0.0006 (6)	0.0003 (6)
O1	0.0444 (7)	0.0296 (6)	0.0148 (5)	0.0054 (5)	−0.0070 (5)	−0.0027 (5)
O2	0.0362 (7)	0.0532 (8)	0.0239 (6)	0.0179 (6)	−0.0045 (5)	−0.0106 (6)
C12	0.0373 (10)	0.0288 (8)	0.0181 (8)	0.0048 (7)	0.0004 (6)	−0.0005 (7)
O3	0.1393 (17)	0.0298 (7)	0.0155 (6)	0.0092 (8)	−0.0021 (7)	−0.0003 (6)
O4	0.0484 (8)	0.0268 (6)	0.0185 (6)	−0.0005 (5)	0.0019 (5)	0.0024 (5)
O5	0.0289 (7)	0.0377 (6)	0.0274 (6)	0.0007 (5)	0.0016 (5)	−0.0031 (5)

### Geometric parameters (Å, °)

**Table:**

Co—O1^i^	2.0574 (12)	C5—C10	1.538 (2)
Co—O1	2.0574 (12)	C6—H6A	0.9700
Co—O5	2.0956 (14)	C6—H6B	0.9700
Co—O5^i^	2.0956 (14)	C7—C8	1.522 (3)
Co—O4^ii^	2.1061 (12)	C7—H7A	0.9700
Co—O4^iii^	2.1061 (12)	C7—H7B	0.9700
C1—C12	1.511 (2)	C8—C9	1.532 (2)
C1—C9	1.533 (2)	C8—C10	1.534 (2)
C1—C6	1.535 (2)	C8—H8A	0.9800
C1—C2	1.540 (2)	C9—H9A	0.9700
C2—C3	1.525 (2)	C9—H9B	0.9700
C2—H2A	0.9700	C10—H10A	0.9700
C2—H2B	0.9700	C10—H10B	0.9700
C3—C7	1.516 (3)	C11—O2	1.237 (2)
C3—C4	1.533 (2)	C11—O1	1.2823 (19)
C3—H3A	0.9800	C12—O4	1.212 (2)
C4—C5	1.532 (2)	C12—O3	1.316 (2)
C4—H4A	0.9700	O3—H1	0.8083
C4—H4B	0.9700	O4—Co^iv^	2.1061 (12)
C5—C11	1.530 (2)	O5—H2	0.8045
C5—C6	1.534 (2)	O5—H3	0.8128
			
O1^i^—Co—O1	180.00 (6)	C6—C5—C10	109.03 (13)
O1^i^—Co—O5	91.39 (5)	C5—C6—C1	110.11 (13)
O1—Co—O5	88.61 (5)	C5—C6—H6A	109.6
O1^i^—Co—O5^i^	88.61 (5)	C1—C6—H6A	109.6
O1—Co—O5^i^	91.39 (5)	C5—C6—H6B	109.6
O5—Co—O5^i^	180.00 (6)	C1—C6—H6B	109.6
O1^i^—Co—O4^ii^	90.51 (5)	H6A—C6—H6B	108.2
O1—Co—O4^ii^	89.49 (5)	C3—C7—C8	109.63 (14)
O5—Co—O4^ii^	88.49 (5)	C3—C7—H7A	109.7
O5^i^—Co—O4^ii^	91.51 (5)	C8—C7—H7A	109.7
O1^i^—Co—O4^iii^	89.49 (5)	C3—C7—H7B	109.7
O1—Co—O4^iii^	90.51 (5)	C8—C7—H7B	109.7
O5—Co—O4^iii^	91.51 (5)	H7A—C7—H7B	108.2
O5^i^—Co—O4^iii^	88.49 (5)	C7—C8—C9	109.50 (15)
O4^ii^—Co—O4^iii^	180.00 (5)	C7—C8—C10	109.98 (15)
C12—C1—C9	112.81 (14)	C9—C8—C10	109.04 (14)
C12—C1—C6	109.83 (13)	C7—C8—H8A	109.4
C9—C1—C6	108.93 (14)	C9—C8—H8A	109.4
C12—C1—C2	106.41 (13)	C10—C8—H8A	109.4
C9—C1—C2	109.37 (14)	C8—C9—C1	109.59 (13)
C6—C1—C2	109.42 (14)	C8—C9—H9A	109.8
C3—C2—C1	109.17 (13)	C1—C9—H9A	109.8
C3—C2—H2A	109.8	C8—C9—H9B	109.8
C1—C2—H2A	109.8	C1—C9—H9B	109.8
C3—C2—H2B	109.8	H9A—C9—H9B	108.2
C1—C2—H2B	109.8	C8—C10—C5	109.71 (13)
H2A—C2—H2B	108.3	C8—C10—H10A	109.7
C7—C3—C2	109.76 (15)	C5—C10—H10A	109.7
C7—C3—C4	109.50 (15)	C8—C10—H10B	109.7
C2—C3—C4	109.95 (15)	C5—C10—H10B	109.7
C7—C3—H3A	109.2	H10A—C10—H10B	108.2
C2—C3—H3A	109.2	O2—C11—O1	122.85 (15)
C4—C3—H3A	109.2	O2—C11—C5	120.66 (14)
C5—C4—C3	109.93 (13)	O1—C11—C5	116.47 (14)
C5—C4—H4A	109.7	C11—O1—Co	125.90 (11)
C3—C4—H4A	109.7	O4—C12—O3	122.98 (16)
C5—C4—H4B	109.7	O4—C12—C1	122.33 (15)
C3—C4—H4B	109.7	O3—C12—C1	114.61 (15)
H4A—C4—H4B	108.2	C12—O3—H1	111.4
C11—C5—C4	111.41 (13)	C12—O4—Co^iv^	131.58 (11)
C11—C5—C6	109.66 (13)	Co—O5—H2	108.0
C4—C5—C6	109.40 (13)	Co—O5—H3	115.6
C11—C5—C10	108.90 (13)	H2—O5—H3	102.6
C4—C5—C10	108.42 (13)		

Symmetry codes: (i) −*x*+1, −*y*+1, −*z*; (ii) −*x*+1, −*y*+1, −*z*+1; (iii) *x*, *y*, *z*−1; (iv) *x*, *y*, *z*+1.

### Hydrogen-bond geometry (Å, °)

**Table:**

*D*—H···*A*	*D*—H	H···*A*	*D*···*A*	*D*—H···*A*
O3—H1···O1^iv^	0.81	1.82	2.6058 (19)	166
O5—H2···O2	0.80	2.07	2.7762 (18)	147
O5—H3···O2^v^	0.81	2.02	2.8334 (18)	175

Symmetry codes: (iv) *x*, *y*, *z*+1;  (v) −*x*+3/2, *y*, *z*−1/2.
